# Image-Based Planning of Minimally Traumatic Inner Ear Access for Robotic Cochlear Implantation

**DOI:** 10.3389/fsurg.2021.761217

**Published:** 2021-11-25

**Authors:** Fabian Mueller, Jan Hermann, Stefan Weber, Gabriela O'Toole Bom Braga, Vedat Topsakal

**Affiliations:** ^1^ARTORG Center for Biomedical Engineering Research, University of Bern, Bern, Switzerland; ^2^Department of Otorhinolaryngology, Head and Neck Surgery, Vrije Universiteit Brussel, Brussels, Belgium; ^3^Department of Otorhinolaryngology, Head and Neck Surgery, University Hospital UZ Brussel, Vrije Universiteit Brussel, Brussels, Belgium

**Keywords:** sensorineural hearing loss, task-autonomous robotics, computer-assisted surgery, image-guided surgery, cochlear implantation, patient-specific planning

## Abstract

**Objective:** During robotic cochlear implantation, an image-guided robotic system provides keyhole access to the scala tympani of the cochlea to allow insertion of the cochlear implant array. To standardize minimally traumatic robotic access to the cochlea, additional hard and soft constraints for inner ear access were proposed during trajectory planning. This extension of the planning strategy aims to provide a trajectory that preserves the anatomical and functional integrity of critical intra-cochlear structures during robotic execution and allows implantation with minimal insertion angles and risk of scala deviation.

**Methods:** The OpenEar dataset consists of a library with eight three-dimensional models of the human temporal bone based on computed tomography and micro-slicing. Soft constraints for inner ear access planning were introduced that aim to minimize the angle of cochlear approach, minimize the risk of scala deviation and maximize the distance to critical intra-cochlear structures such as the osseous spiral lamina. For all cases, a solution space of Pareto-optimal trajectories to the round window was generated. The trajectories satisfy the hard constraints, specifically the anatomical safety margins, and optimize the aforementioned soft constraints. With user-defined priorities, a trajectory was parameterized and analyzed in a virtual surgical procedure.

**Results:** In seven out of eight cases, a solution space was found with the trajectories safely passing through the facial recess. The solution space was Pareto-optimal with respect to the soft constraints of the inner ear access. In one case, the facial recess was too narrow to plan a trajectory that would pass the nerves at a sufficient distance with the intended drill diameter. With the soft constraints introduced, the optimal target region was determined to be in the antero-inferior region of the round window membrane.

**Conclusion:** A trend could be identified that a position between the antero-inferior border and the center of the round window membrane appears to be a favorable target position for cochlear tunnel-based access through the facial recess. The planning concept presented and the results obtained therewith have implications for planning strategies for robotic surgical procedures to the inner ear that aim for minimally traumatic cochlear access and electrode array implantation.

## Introduction

Robotic cochlear implantation is emerging with the objective to standardize surgical outcomes for patients with sensorineural hearing loss. It is designed to conduct cochlear access relying on image-based accurate surgical planning and activity using a sensor- and image-guided robotic system (1–5). The keyhole access to the cochlea (*cochlea*) is obtained through a robotically drilled tunnel from the lateral surface of the mastoid through the facial recess (*sinus facialis*) to the round window (*fenestra cochleae*) (RW) of the cochlea. This robotic activity is considered task autonomous, according to the definition of autonomy levels for medical robotics as introduced by Yang et al. (6). The objective of the robotic task presented herein is to standardize minimally traumatic access to the cochlea. In this context, a procedure is considered minimally traumatic if no mechanical trauma occurs during robotic activity; a condition that is met if the anatomical and functional integrity of critical structures of the middle ear (*auris media*) and inner ear (*auris interna*) remain preserved. The importance of protecting critical intra-cochlear structures for residual hearing preservation during inner ear access and electrode array insertion is a widely discussed research topic. There are high expectations that a robotic approach could reduce trauma to the cochlea. However, it remains to be proven whether this is a sufficient condition for preserving residual hearing; biological factors also need to be investigated.

For cochlear implantation surgery, it is critical to have a precise anatomical knowledge of the region of the RW including its anatomical microenvironment. The RW niche (*fossula fenestrae cochleae*), is an open cave-like area with an overhanging oblique ridge from the promontory consisting of a posterior pillar (*postis posterior*), a tegmen (*tegmen*) and an anterior pillar (*postis anterior*). The superior part, which resembles a canopy and covers the round window membrane (*membrana tympani secundaria*) (RWM), is referred to as the canonus (*canonus fossulae fenestrae cochleae*) (7–9). The RWM which is embedded in the RW niche, covers the entrance to the scala tympani and has a complex variable conical shape with a posterior portion close to the osseous spiral lamina (10). This distance increases from about 0.1 mm to about 1 mm, as does the width and height of the scala tympani as one moves anteriorly and inferiorly to the center of the RW (11). The scala tympani, the favored intra-cochlear lumen for implant placement, can be accessed through a RW or extended RW approach or a RW-related cochleostomy (12, 13). A favorable trajectory directed into the scala tympani, without targeting the osseous spiral lamina and the lateral wall of the basal portion, must pass through the canonus of the niche (14). Removal of the canonus (canonectomy) or creation of an opening in the canonus (canonostomy) may cause trauma to the hook region, where the osseous spiral lamina, the spiral ligament and the basilar membrane fuse (10). To avoid damage to the basilar membrane and mitigate a reduction of the hair cell and nerve fiber population, it is important to anatomically preserve the osseous spiral lamina (15).

In conventional cochlear implantation surgery, the surgeon removes the complete superior part (canonectomy) to create a visual exposure of the RWM for orientation during insertion of the cochlear implant electrode. This procedure is conducted at the limit of human tactile feedback and sensory capabilities (16). Therefore, trauma may result from direct mechanical damage to the anatomy caused by the hand-guided tool or indirectly from the high induced sound pressure within the cochlea (17). Efforts have been made to provide a more consistent approach minimizing induced trauma on the hearing organ with the use of a force guided controlled tool or a robotic system (18–24). All of these developed approaches aimed for robust controlled penetration of the outer bone shell of the cochlea without penetration of the RWM. With the robotic approach, the opening of the canonus could be reduced to a circle with a diameter of 1.0 mm (canonostomy), allowing the electrode array to be passed through the drilled tunnel without visual exposure of the entire RWM (5). This surgical technique allows removal of drill debris prior to electrode insertion and minimizes induced disturbance and sound pressure on the cochlea (17, 25, 26). Regardless of the method, it is generally concluded, that the RWM must be preserved during the canonectomy or canonostomy to minimize trauma to the cochlea (13, 27). Additionally, it is concluded, that the ideal insertion trajectory should align with the centerline of the scala tympani to prevent damage to intra-cochlear structures during electrode array insertion (23, 28). While there is consensus on the optimal position for accessing the RW in conventional cochlear implantation surgery, this has not been adequately studied in tunnel-based robotic cochlear implantation (13).

There are several factors affecting the optimal target position and trajectory orientation in robotic cochlear implantation. This includes the size and shape of the facial recess, the variable anatomy of the RW including the basal portion of the cochlea, and the size and orientation of the scala tympani (29, 30). In addition, the dimensions of the surgical tools and the accuracy of the robotic system have an important role in limiting the direction of entry into the scala tympani and the size of the feasible target region (31). Recent research suggested a target position central or inferiorly to the center of the RWM with the optimal trajectory defined to minimize the cochlear in- and out plane angle (13, 23). The in- plane angle is the offset between the optimal and the ideal trajectory that delineates alongside the lateral wall of the basal turn for a given target position. However, this definition of an optimal target position does not take into account the complex anatomy of the RW and the intra-cochlear hook region in intra-operative planning, and aims only for reliable electrode insertion within the scala tympani. Due to limited clinical imaging modalities, the RW and the bony cochlear wall remain the only consistent landmarks in intra-operative planning. To standardize trajectory planning, more precise planning parameters and criteria for inner ear access need to be introduced. Ideally these are expressed in terms of anatomical and structural properties of the RW and the bony cochlear wall to allow a consistent and accurate characterization of an optimal trajectory with clinical imaging modalities.

The aim of this work was to evaluate an optimal trajectory to the inner ear in tunnel based robotic cochlear implantation taking into account the complex RW anatomy and its anatomical microenvironment. A set of complementary hard and soft constraints for middle ear and inner ear access were proposed to calculate an optimal trajectory solution space. The hard constraints ensure, that the trajectory passes through the facial recess and maintains a safe distance to critical middle ear and intra-cochlear structures. In parallel, the soft constraints for the inner ear access aim to minimize the angle of cochlear approach, minimize the risk of scala deviation and maximize the distance to critical intra-cochlear structures. This approach of trajectory planning is defined as a multi-criteria constraint optimization problem. The solution space was evaluated to derive possible implications for tunnel-based robotic access to the inner ear.

## Materials and Methods

### Adaption of the OpenEar Library

**Figure 1:**
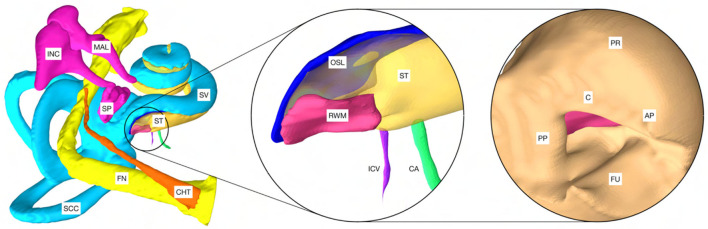
Model of a cochlea of the right human ear based on the OpenEar dataset. CHT, chorda tympani; FN, facial nerve; SCC, semicircular canals; MAL, malleus; INC, incus; SP, stapes; ST, scala tympani; SV, scala vestibule. The magnification in the center shows the extensions made to the model: RWM, round window membrane; OSL, osseous spiral lamina; ICV, inferior cochlear vein; CA, cochlear aqueduct. Right: PP, posterior pillar; C, canonus; AP, anterior pillar; FU, fustis; PR, promontory. Note: For visualization purposes, the model of the external ear canal (*meatus acusticus externus*) was excluded.

The planning analysis conducted in this study was based on the OpenEar library consisting of the data set of eight human temporal bones (five right side, three left side) (32). Each dataset is based on a combination of multimodal imaging including cone beam computed tomography and micro-slicing with the corresponding segmentation of inner ear compartments, middle ear bones, tympanic membrane, relevant nerve structures, blood vessels, and the temporal bone (33). For this study, the segmentation of the dataset was extended to include relevant inner ear structures that were discernible by the micro-slicing reconstruction method, these include the RWM, osseous spiral lamina, inferior cochlear vein, and the cochlear aqueduct. Due to the limited image quality available, the osseous spiral lamina, the basilar membrane, and the secondary spiral lamina could not be reliably separated during segmentation and were combined in the model of the osseous spiral lamina (15). All segmentations were carried out with 3D Slicer, an open source software platform for medical image informatics, image processing, and three-dimensional visualization (http://www.slicer.org) (34). The final output was a library consisting of eight datasets with the aforementioned extension made to the model (Figure 1). For comparability, the naming of the cases in this work was adopted from the OpenEar library.

### Hard Constraints for Trajectory Planning

An approach was developed to automatically plan a trajectory to the RW that fulfills anatomical safety margin constraints and aims to optimize the soft constraints for inner ear access. For safety related considerations, hard constraints were introduced to maintain a safe predefined distance to all structures at risk (Table 1). The anatomical safety margins were adopted from the otological planning software OTOPLAN (Version 1.5.0, CASCINATION AG, Switzerland). These are to be understood as the minimum accepted distances from the anatomy at risk to the surgical drill. In this study, the tool set of the HEARO robotic system (CASCINATION AG, Switzerland) consisting of the HEARO Step Drill Bit 1.8 mm for middle ear access (∅ 1.8–2.5 mm) and the HEARO Diamond Burr for inner ear access (∅ 1.0 mm) were used to calculate the safety margins. For this particular robotic system, the safety margins are fulfilled if the tool has a minimum distance of 0.4 mm to the facial nerve and 0.3 mm to the chorda tympani and all other structures at risk (Table 1) (35, 36). There are no reference values available for safe distance to intra-cochlear structures. In this work, the safety margin to intra-cochlear structures was constrained to 0.2 mm. This value was concluded to be adequate based on the current reported accuracy of the robotic system (0.15 mm, *SD* = 0.08) (2). However, an additional soft constraint as introduced later, aimed to increase this intra-cochlear safety margin.

**Table 1:** Middle ear and inner ear access hard and soft constraints.

**Hard constraints**	**Access**	**Anatomy**	**Constrained value**	**Priority**
Safety margin	Middle ear	Facial nerve	0.4 mm	-
		Chorda tympani	0.3 mm	
		Incus		
		Malleus		
		Stapes		
		External auditory canal		
	Inner ear	Osseous spiral lamina	0.2 mm	
		Inferior cochlear vein		
		Cochlear aqueduct		
**Soft constraints**	**Access**	**Anatomy**	**Objective**	**Priority**
Angle of cochlear approach φ	Inner ear	–	Minimize φ	20%
RWM coverage ratio r		Round window membrane	Maximize r	60%
Intra-cochlear distance d		Osseous spiral lamina	Maximize d	20%
		Inferior cochlear vein		
		Cochlear aqueduct		

### Target Region and Candidate Trajectories

The RW approach is considered the best approach for minimally traumatic access to the scala tympani. Therefore, the lateral RWM area was defined as the potential target region for trajectory planning. In a first step, the RWM target region was sampled and constrained by potential target positions that have a sufficient distance to all relevant intra-cochlear structures. A distance of 0.7 mm was determined based on the diameter of the burr (∅ 1.0 mm) together with the constrained distance of 0.2 mm to the structures. Therefore, all target positions on the RWM not fulfilling a minimum distance of 0.7 mm to the closest intra-cochlear structure were excluded from the target region. In a further step, all possible and reasonable trajectory orientations for the remaining target region were generated in a uniformly sampled volume. These trajectories were further decimated by the trajectories that did not meet the hard constraints for access to the middle ear and inner ear (Table 1). The remaining trajectories were designated as candidate trajectories and considered for further investigation.

### Soft Constraints for Inner ear Access

**Figure 2:**
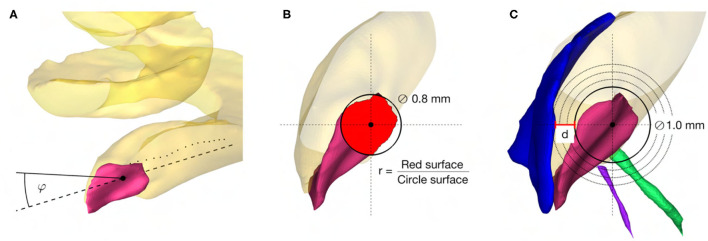
**(A)** Angle of cochlear approach constraint minimizing the angle φ in three-dimensional space between the linear approximation (−−) of the scala tympani centerline (··) and the candidate trajectory (–). **(B)** RWM coverage ratio constraint maximizing the ratio r between the projection of the electrode (∅ 0.8mm) on the RWM (red area) and the electrode cross-sectional area. **(C)** Intra-cochlear structure distance constraint maximizing the distance d to the closest intra-cochlear structure, here the osseous spiral lamina.

The following soft constraints were introduced based on the current knowledge of the anatomy, experience, and findings in planning and execution of robotic inner ear access (Figure 2).

#### Minimum Angle Between the Trajectory and the Scala Tympani

The angle of cochlear approach φ is the minimum angle in three-dimensional space between the candidate trajectory and the linear approximation of the scala tympani centerline in the RW periphery (Figure 2A). This angle can be further decomposed in the in-plane and the out-plane angle as commonly used in literature to depict deviations from the ideal trajectory in two planes (13, 23).

#### Maximum RWM Coverage Ratio

The coverage ratio *r* is the maximum ratio between the cross-sectional area of the electrode projected onto the RWM along the candidate trajectory and the electrode cross-sectional area (Figure 2B). This soft constraint accounts for the offset of the trajectory from the centerline of the scala tympani and is an indicator of proximity to the RW antero-inferior border, where in most cases the sharp bony crest of the RW (*crista fenestrae cochleae*) is localized. This crest of the RW is a potential obstacle for adequate access to the scala tympani (10, 37, 38) .

#### Maximum Distance to Critical Intra-Cochlear Structures

The distance *d* is the maximum Euclidean distance from the tool to the closest critical intra-cochlear structure for the candidate trajectory (Figure 2C). This allows the hard-constrained minimal safety distance of 0.2 mm to be increased in order to reduce the risk of potential mechanical trauma to intra-cochlear structures, especially considering the accuracy of the robotic system.

### Target and Trajectory Solution Space

**Figure 3:**
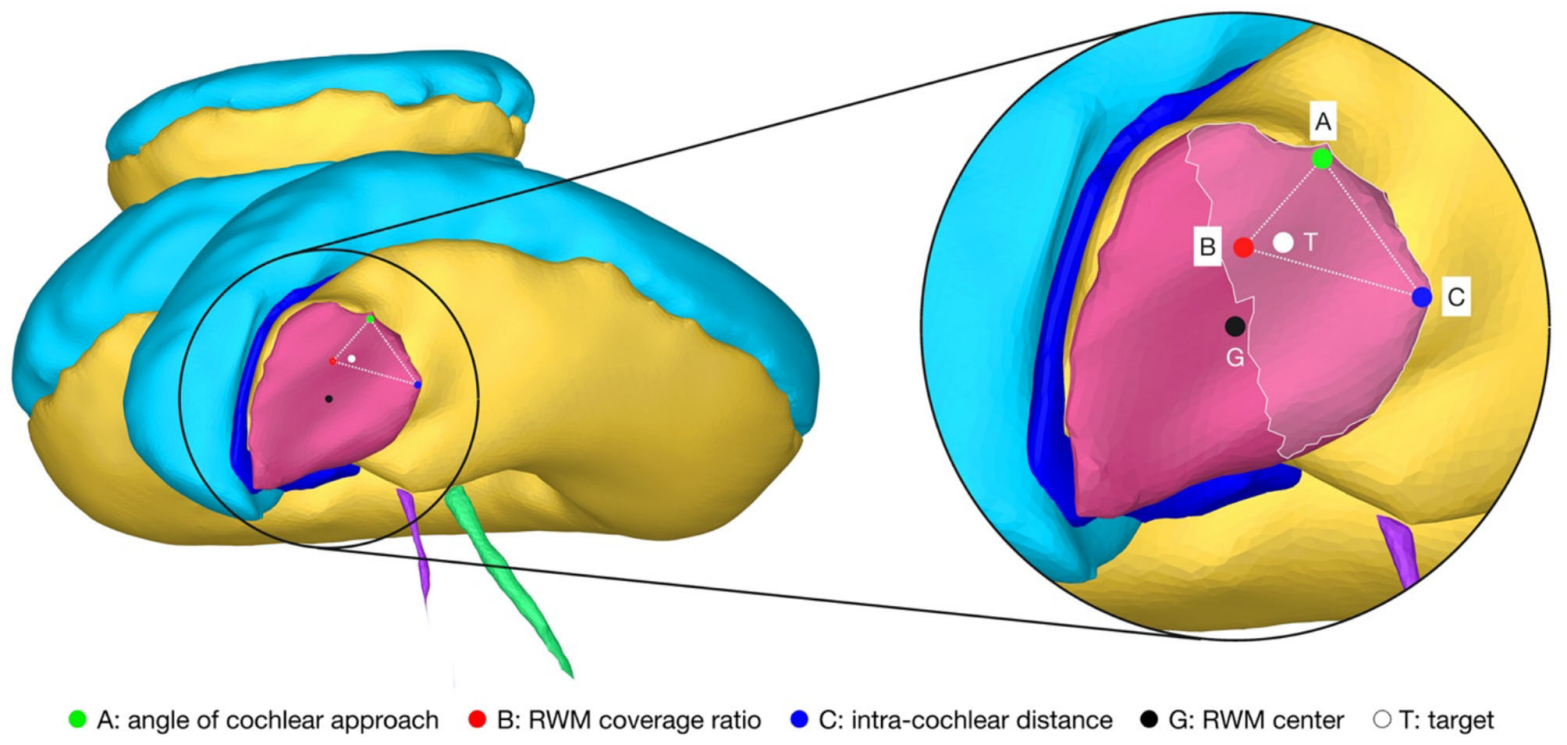
Cochlea of the right ear as seen along the line from the center of the RWM (G) to the apex of the cochlea that is parallel to the cochlear plane. The magnification on the right shows the target region (highlighted area) and the Pareto-optimal target solution space (··) on the RWM. The optimal solution space is spanned by the individual best solutions A, B and C of each soft constraint. T, optimal target position with user-defined priorities; G, geometric center of the RWM.

For the introduced hard and soft constraints an optimal solution space of target positions on the RWM with the corresponding trajectory orientation was calculated with the set of candidate trajectories. The optimal target solution space is spanned by the optimal solutions of the three soft constraints, termed the basic solutions (Figure 3). All solutions in the target position solution space on the RWM are Pareto-optimal. A Pareto optimum is a state in which it is not possible to improve one soft constraint without at the same time having to worsen another. An optimal trajectory orientation was assigned to each individual target position. In addition to the Pareto optimal solution space, a final trajectory was calculated with the user-defined priorities listed in Table 1. The algorithms and the computations were implemented and conducted in MATLAB 2019b using the Parallel Computing Toolbox (39).

### Inner Ear Access Parameterization and Virtual Canonostomy

**Figure 4:**
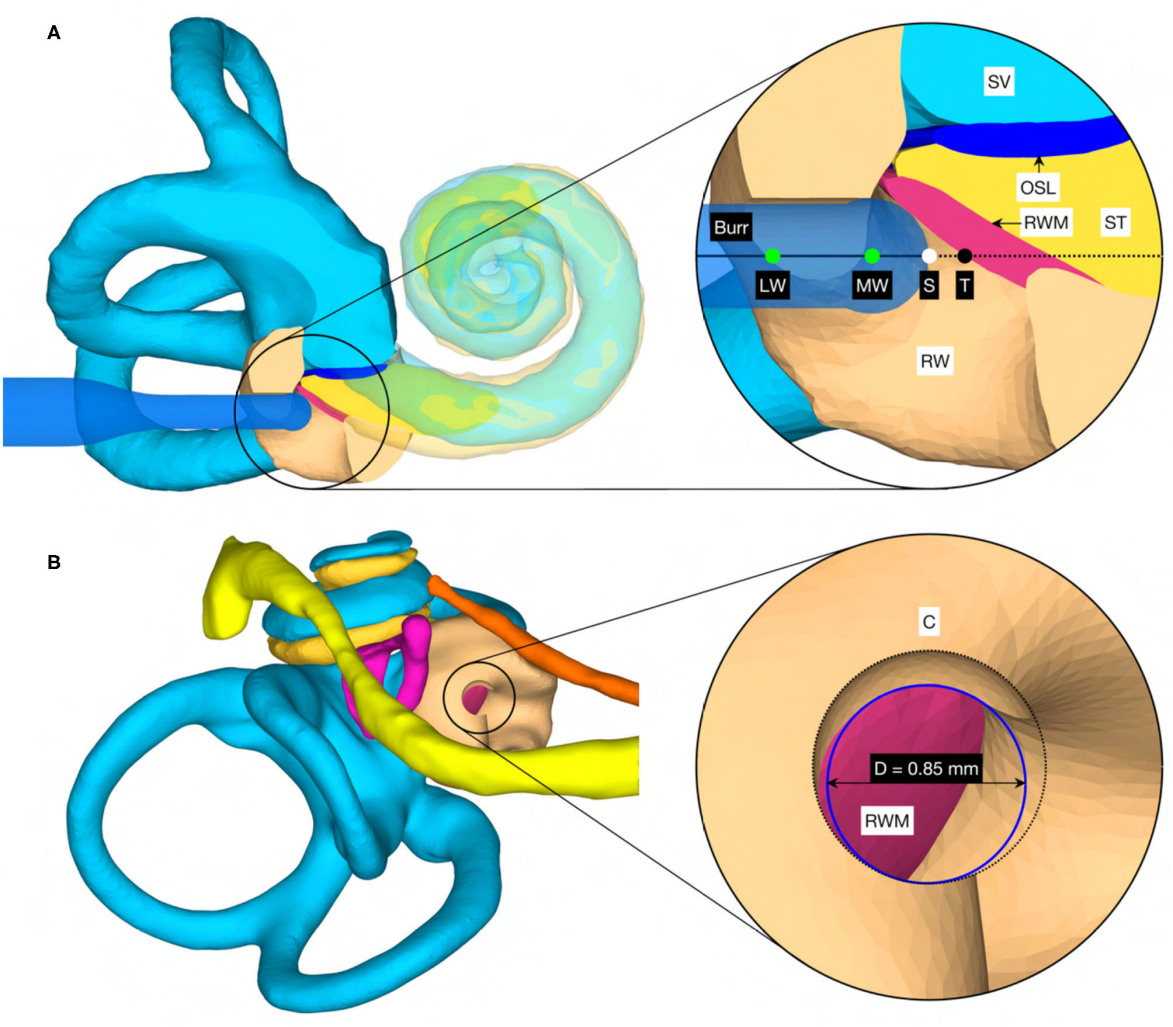
**(A)** Cross-section through the cochlea of a right ear in the cochlear trajectory plane with the tool at the milling stop position. This plane is parallel to the trajectory and as parallel as possible to the cochlear plane while going through the milling stop point. The magnification on the right shows a cross-section trough the intra-cochlear anatomy with the axially defined inner ear access parameters. LW, lateral wall; MW, medial wall; S, stop depth; T, target position. **(B)** View of the cochlea along the trajectory with the virtual opening and the medial opening diameter D of the canonostomy.

In addition to the target position and orientation of the trajectory, parameters were also defined axially along the trajectory to define the surgical procedure of the canonostomy in the RW niche. These parameters include the lateral and medial wall of the canonus and the milling stop depth. The lateral wall was defined as the position where the tool first contacts the canonus when approaching laterally along the trajectory, while the medial wall was defined as the posterior border of the RWM. The milling stop depth was defined as the position where the tool first contacts the RWM laterally (Figure 4A). According to this definition, the lateral wall and the milling stop depth depend on the geometric shape of the burr. The tip of the milling burr is composed of a diamond-coated hemisphere with a cylindrical extension and has a total cutting length of 4 mm with a diameter of 1 mm. A virtual canonostomy was created through a Boolean subtraction of the milling burr from the canonus, with the milling burr positioned co-axial to the trajectory at the depth of the milling stop depth (Figure 4B). The maximum opening diameter of the virtual canonostomy was defined by the maximum circle size that fits axially projected into the opening of the medial wall of the canonus.

## Results

### Target and Trajectory Solution Space

**Figure 5:**
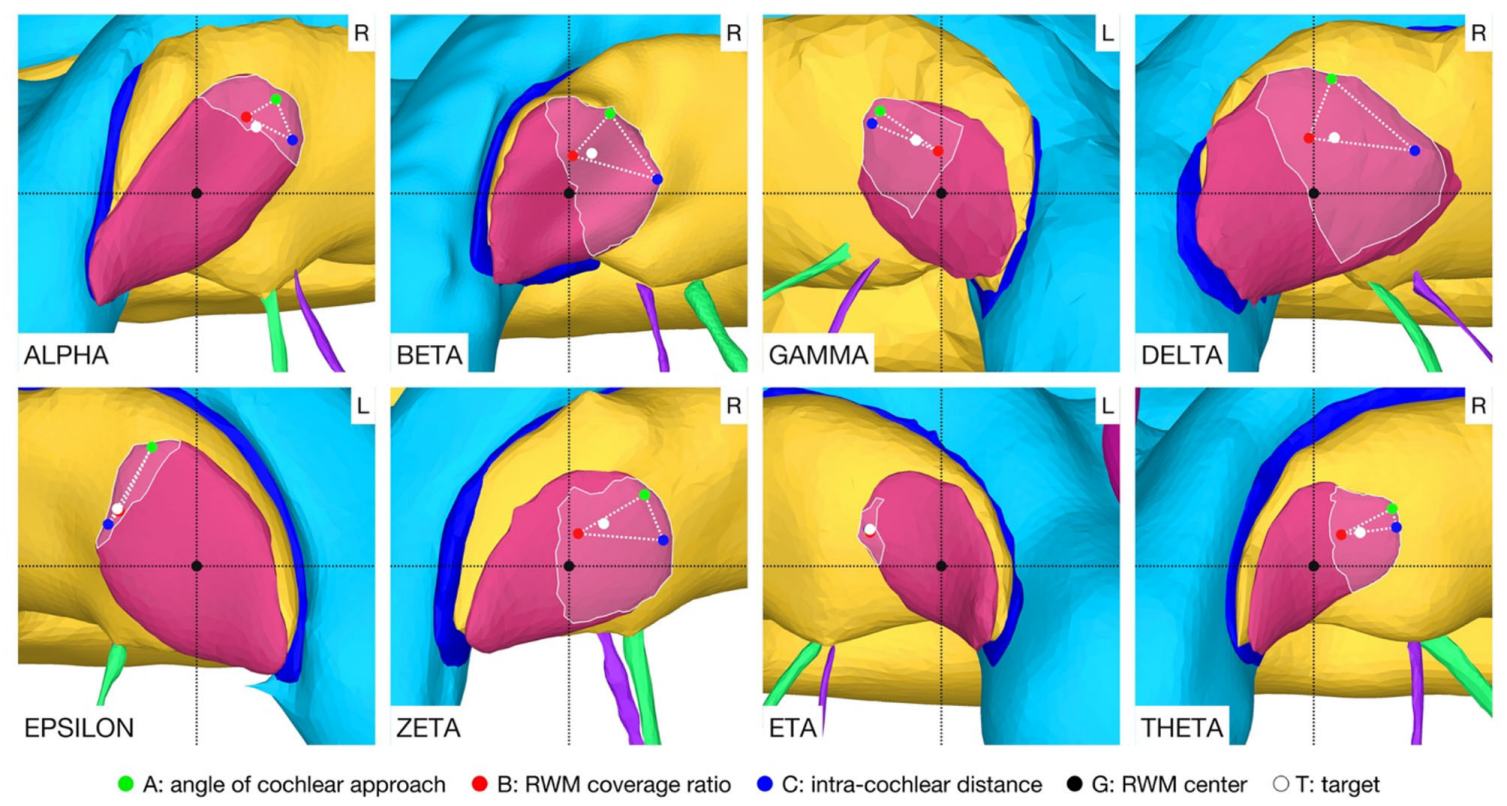
Feasible target region (highlighted area) and the optimal target solution space (··) on the RWM. The optimal solution space is spanned by the individual best solutions A, B, and C of each soft constraint. L, left side cochlea; R, right side cochlea.

A target and trajectory solution space was successfully calculated for each case based on the introduced middle ear and inner ear access constraints. The feasible target region on the RWM includes all target positions for which a trajectory exists that satisfies the hard constraints. This domain was further confined by the optimal target solution space wherein all solutions are Pareto-optimal with respect to the soft constraints (Figure 5). Additionally, a target position was calculated based on the user-defined priorities. In most cases, with the exception of the cases EPSILON and ETA, the target position was close to, and approximately halfway along the line directed from the antero-inferior border to the center of the RWM. It was observed that the best target position for maximizing the angle of cochlear approach constraint was the antero-inferior border of the RWM, while for the intra-cochlear structure distance constraint, this position was more inferior. As expected from the geometric arrangement of the RWM and the trajectory orientation, the best position to maximize the RWM coverage ratio constraint was closer to the center of the RWM. The size of the feasible target region ranged from 0.066 to 1.566 mm^2^ with an average area of 0.604 mm^2^ (*SD* = 0.485). The cases EPSILON and ETA had a very limited feasible target region and consequently only a local concentrated region for optimal target positions.

**Figure 6:**
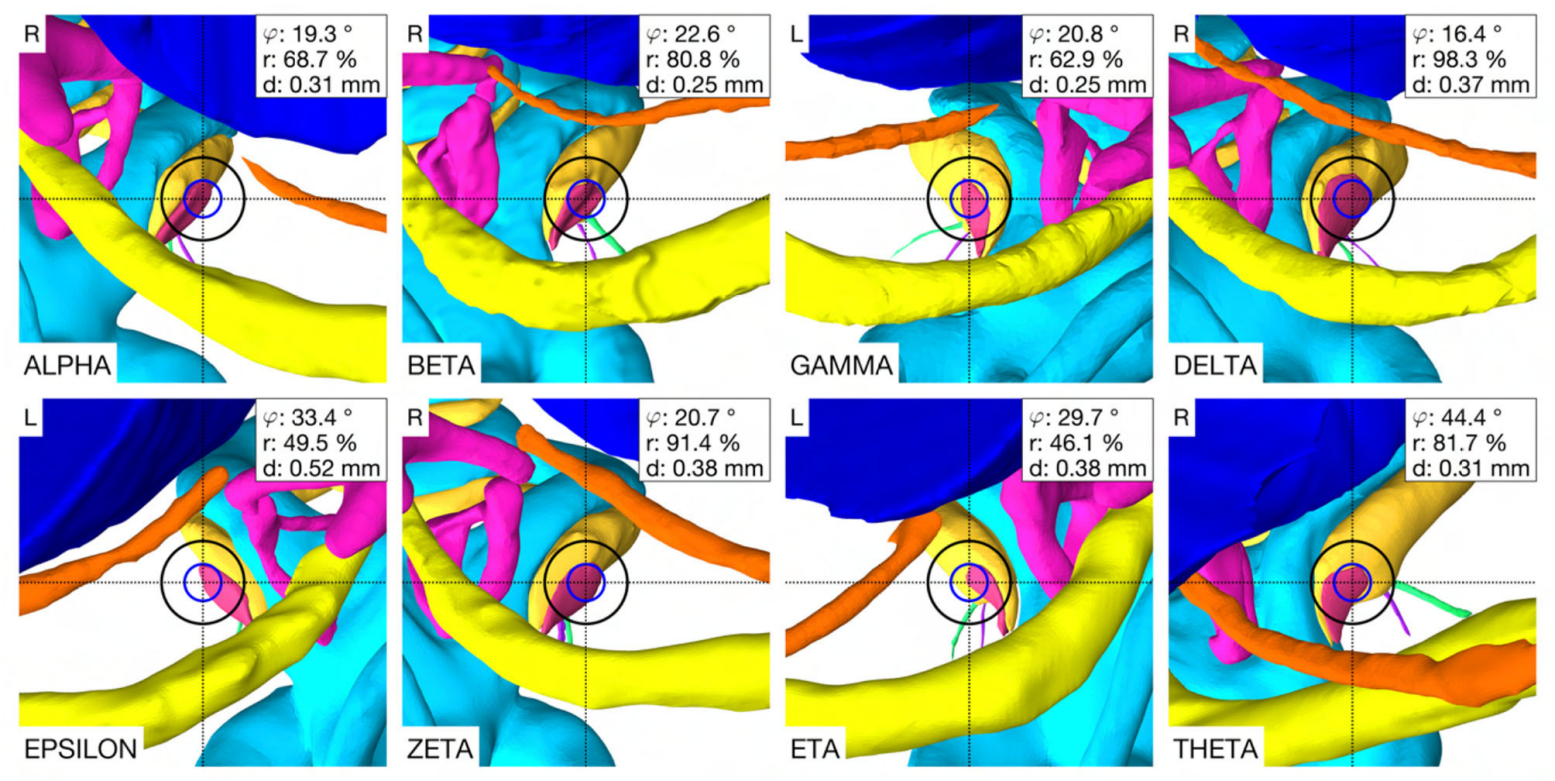
Optimal trajectory with the user-defined priorities. Each case shows the view along the trajectory to the RWM with the corresponding soft constraint optimization value φ, r and d. Blue circle, diameter of the electrode (∅ 0.8 mm), black circle, tool diameter at the depth of the facial recess (∅ 1.8 mm), L, left side cochlea; R, right side cochlea.

**Figure 7:**
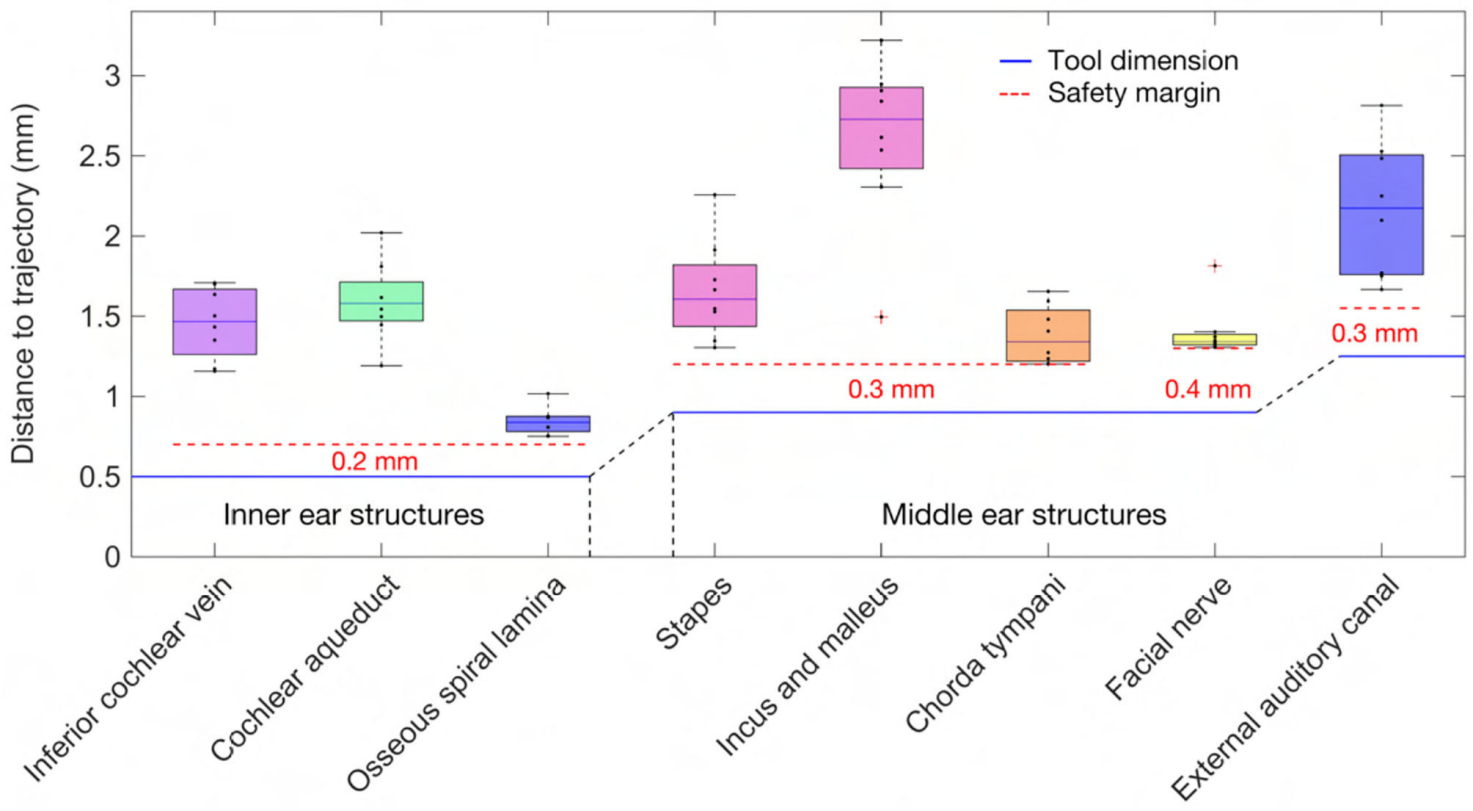
Distance of the anatomy to the trajectory together with the dimension of the tool and the constrained safety margins to the critical middle ear and inner ear structures.

In the case THETA, a facial recess trajectory orientation could not be calculated as the facial recess was too narrow and a collision with the facial nerve or the chorda tympani would have been inevitable (Figure 6). In all other cases, the trajectory calculated with the user-defined priorities fulfilled all safety margins for access to the middle ear and inner ear (Figure 7). The distances to the facial nerve were very close to the constrained safety margin and ranged from 0.405 to 0.503 mm with an average value of 0.443 mm (*SD* = 0.034), excluding the case THETA. In all cases, the shortest distance to the intra-cochlear structures was the distance to the osseous spiral lamina and ranged from 0.251 to 0.516 mm with an average value of 0.350 mm (*SD* = 0.092). In general, with a larger feasible target region, mainly related to a wider facial recess, a higher optimality of the soft constraint values was achieved. In particular, for the cases EPSILON and ETA, which had a limited feasible target region, only a low optimization value was obtained for the angle of cochlear approach φ and the RWM coverage ratio r.

### Inner Ear Access Parameterization and Virtual Canonostomy

**Figure 8:**
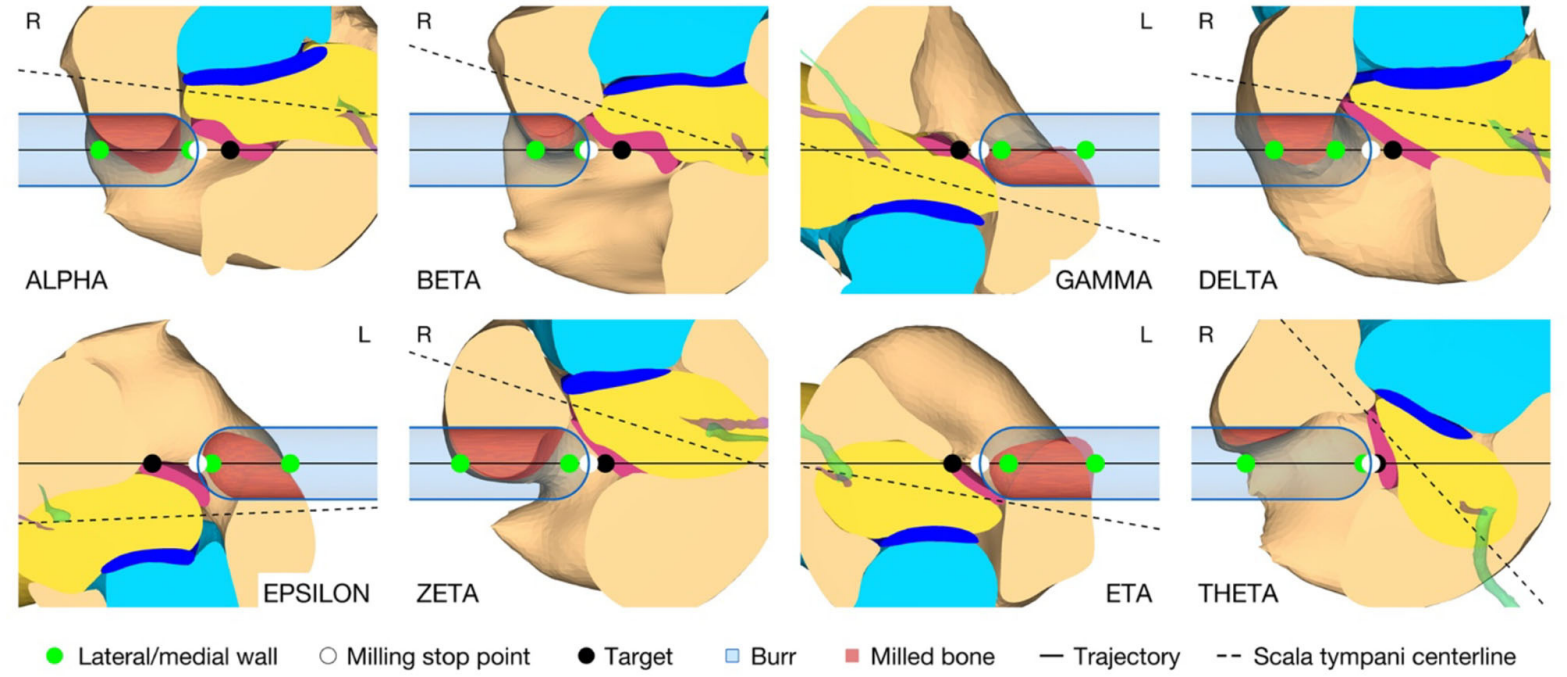
Cross-section through the RW along the cochlear trajectory plane showing the intra-cochlear structures, the burr at its milling stop position, and the surgical parametrization of the canonostomy. L, left side cochlea; R, right side cochlea.

**Figure 9:**
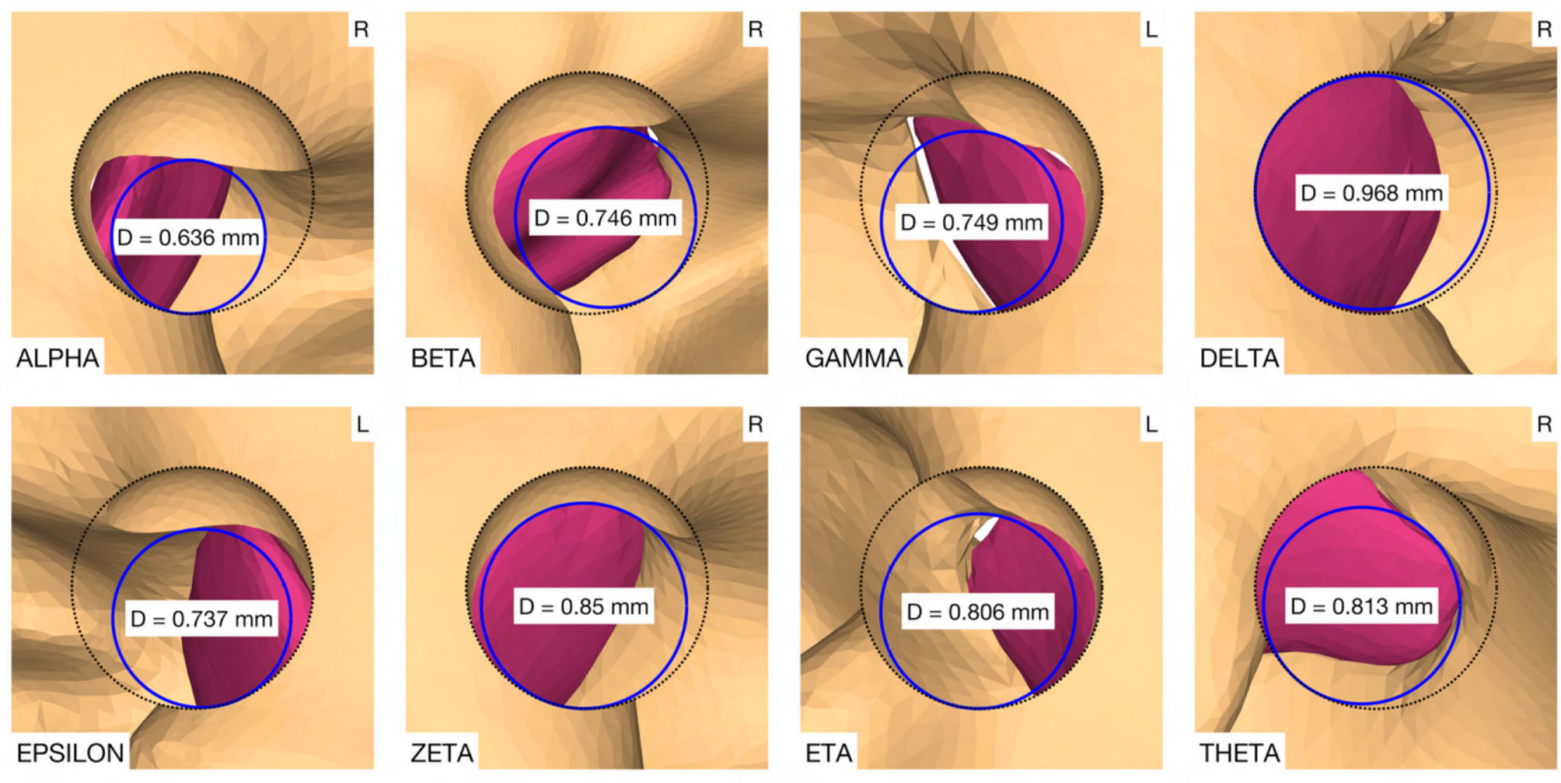
Canonostomy from a trajectory view. D, medial opening diameter of the canonus. L, left side cochlea; R, right side cochlea.

The virtual surgical procedure of creating an access hole in the canonus based on the aforementioned inner ear access parameterization was performed for all cases (Figure 8). It could be observed that a safe distance to the osseous spiral lamina was maintained and that the RWM was not perforated as expected according to the definition of the milling stop depth. Therefore, the intra-cochlear structures were not in contact with the milling burr during the virtual canonostomy. In addition to the angle of cochlear approach, a lateral offset of the trajectory from the scala tympani centerline was observed in most cases. The measured circular opening diameter at the medial wall of the canonus ranged from 0.636 to 0.968 mm with an average value of 0.788 mm (*SD* = 0.097) (Figure 9).

## Discussion

In conventional cochlear implantation surgery, there is consensus that an electrode insertion vector from postero-superior to antero-inferior to the RWM potentially avoids scala deviation and preserves the osseous spiral lamina and the basilar membrane (30, 40). In recent robotic cochlear implantation, the target position was placed in the center of the RW and planning of a trajectory through the facial recess with minimal cochlear in- and out plane angles was considered as an optimal insertion trajectory (13, 23). This definition did not take into account the close proximity to intra-cochlear structures during planning due to insufficient clinical imaging modalities and primarily aimed for a reliable electrode insertion within the scala tympani. During image-based clinical planning, intra-cochlear structures cannot be identified and segmented, therefore their position and shape must be estimated based on their local relationship to the RW and the bony cochlear wall, the only consistent landmarks.

This work introduced additional inner ear access constraints for trajectory planning and used high resolution anatomical models to account for the imaging limitations of the clinical approach. The soft constraints were defined based on in-depth knowledge of the anatomy, experience and findings regarding planning and execution of robotic inner ear access and manual electrode insertion. Due to the definition of multiple criteria and the nature of the spatial relationship between the anatomical structures, there was no unique solution for an optimal target position and trajectory orientation. Rather, there was an entire solution space of optimal trajectories that could be explored with the adaption of priorities that affect the individual soft constraints of the inner ear access. The results showed that the size and shape of the feasible target region was highly variable. This could be explained with the high variability of the shape and size of the RW and the spatial relationship between the basal turn and the facial recess (38, 41, 42). The size of the facial recess directly limits the possible orientations of the trajectory and thus the accessibility to the scala tympani. Therefore, cases with a narrow facial recess had either no solution or minimal freedom in target and trajectory optimization, as observed in the cases EPSILON and ETA. This problem could be addressed by using surgical tools with a smaller diameter, for example ∅ 1.4 mm instead of ∅ 1.8 mm at the level of the facial recess. The difficulty here, however, would be the development of electrode guide tubes that could be placed in smaller diameter tunnels, which are mostly needed as insertion aid to avoid kinking in the usually highly aerated mastoid bone (mastoid antrum, mastoid cells; *antrum mastoideum, cellulae mastoideae*) (4).

The results of this work showed, that there is a clear tendency that a position between the antero-inferior border and the center of the RWM may prove to be the optimal position for cochlear tunnel based access. This target position would potentially avoid damage to critical inner ear and middle ear structures while providing minimal insertion angles and a sufficient cochlear opening for electrode insertion. In some analyzed cases, the measured diameter of the medial opening of the canonus was slightly smaller than the diameter of most existing implants at the depth of the RW (∅ 0.8 mm). However, it is assumed that the thin layer of remaining bone shell could be easily removed by the surgeon during the opening of the RWM and may also contribute to a better fixation of the electrode in the RW niche. An extremely small or narrow shaped RW with a diameter smaller than the diameter of the cochlear implant array could make a minimally traumatic access difficult because an enlarged RW approach would be required. In addition, the sharp bony crest of the RW could be a potential obstacle for soft insertion of the electrode array. The corresponding trajectory orientation could result in bending of the electrode array at the antero-inferior margin of the RW niche and the bony crest could damage the electrode array during insertion or over time. Additional removal of bone in this area to allow adjustment of the insertion vector and to reduce mechanical resistance during insertion should be avoided, as the close proximity to the hook region could potentially traumatize the cochlea and result in loss of residual hearing (38). Therefore, the implications of the proposed target position and trajectory orientation for minimally traumatic electrode array insertion need to be investigated experimentally. It would also be conceivable that patient-specific access priorities could be introduced in clinics. In patients with profound hearing loss, it would be less important to preserve specific inner ear structures. Planning priorities could be adjusted to focus on depth and placement quality of the electrode, and only in a patient seeking preservation of residual hearing, priorities could be set on the minimally traumatic approach.

The planning concept presented in this work was not based on image data available in routine clinical practice as the current computed tomography technology used in clinics does not provide the necessary image resolution to detect intra-cochlear structures. Consideration must also be given to the fact that the calculation of the entire trajectory solution space is computationally expensive and time consuming, and therefore is not an ideal approach for intra-operative planning. Despite these considerations, the planning concept introduced and the information obtained therewith are helpful and guiding for the planning strategies in future implementations. Current otological planning software is already capable of intra-operatively segmenting the bony anatomy of the RW and modeling the RWM. Moreover, it could be concluded from the results that the calculation of the optimal trajectory solution space can be limited to the antero-inferior region of the RWM. Therefore, it might be possible to already implement planning strategies that allow for potentially less traumatic robotic access to the cochlea. However, the applicability of the planning concept in clinical image-based planning and the efficacy of the corresponding surgical approach for minimally traumatic cochlear access need to be investigated in further studies.

## Conclusion

Incorporating the introduced hard and soft constraints for the inner ear access during trajectory planning, a tendency could be identified that a position between the antero-inferior border and the center of the RWM could be a favorable target position for tunnel-based cochlear access. The planned trajectories were compatible with the middle ear access, would potentially avoid damage of critical intra-cochlear structures during robotic execution, and would allow implantation with minimal insertion angles and risk of scala deviation. The planning concept presented, as well as the findings obtained therewith, have implications for planning strategies for tunnel-based robotic surgical procedures to the inner ear that aim for minimally traumatic cochlear access and electrode array implantation.

## Data Availability Statement

The raw data supporting the conclusions of this article will be made available by the authors, without undue reservation.

## Author Contributions

FM created the OpenEar library extension, developed and evaluated the planning concept, and is the primary author of the manuscript. VT, JH, GO'T, and SW contributed with their scientific advice. All authors reviewed the manuscript and approved the submitted version.

## Funding

This work was supported by the Swiss National Science Foundation SNF (Project 176007).

## Conflict of Interest

SW is cofounder, shareholder, and chief executive officer of CASCINATION AG (Bern, Switzerland), a spin-off company from our university that commercializes the robotic cochlear implantation technology. The remaining authors declare that the research was conducted in the absence of any commercial or financial relationships that could be construed as a potential conflict of interest.

## Publisher's Note

All claims expressed in this article are solely those of the authors and do not necessarily represent those of their affiliated organizations, or those of the publisher, the editors and the reviewers. Any product that may be evaluated in this article, or claim that may be made by its manufacturer, is not guaranteed or endorsed by the publisher.
